# Highly efficient photocatalytic degradation of methylene blue by PoPD/TiO_2_ nanocomposite

**DOI:** 10.1371/journal.pone.0174104

**Published:** 2017-03-22

**Authors:** Chuanxi Yang, Ming Zhang, Wenping Dong, Guanwei Cui, Zongming Ren, Weiliang Wang

**Affiliations:** 1 College of Geography and Environment, Shandong Normal University, Jinan, People's Republic of China; 2 College of Resources and Environmental Sciences, China Agricultural University, Beijing, People's Republic of China; 3 NJTECH Environment Technology Co., Ltd, Nanjing, People's Republic of China; 4 Shandong Academy of Environmental Science and Environmental Engineering Co., Ltd, Jinan, People's Republic of China; 5 College of Chemistry, Chemical Engineering and Materials Science, Key Laboratory of Molecular and Nano Probes, Ministry of Education, Shandong Normal University, Jinan, People's Republic of China; 6 Institute of Environment and Ecology, Shandong Normal University, Jinan, People's Republic of China; Institute of Materials Science, GERMANY

## Abstract

The poly-o-phenylenediamine (PoPD)/TiO_2_ nanocomposite was successfully synthesized via ‘in situ’ oxidative polymerization method. The modified photocatalysts were characterized by BET, scanning electron microscopy (SEM), transmission electron microscopy (TEM), X-ray diffraction (XRD), Fourier-transform infrarad spectra (FT-IR), thermogravimrtic analysis (TGA), X-ray photoelectron spectroscopy (XPS), Ultraviolet-visible diffuse reflectance spectrum (UV-Vis DRS) and Photocurrent Test. The results showed that the PoPD exists on the surface of TiO_2_, the presence of PoPD does not impact on the lattice structure and grain size of TiO_2_, and the presence of PoPD enhances the visible response and photoelectric property. The photocatalytic degradation of methylene blue (MB) was chosen as a model reaction to evaluate the photocatalytic activities of TiO_2_ and PoPD/TiO_2_. The optimal preparation condition was the molar ratio of oPD to TiO_2_ = 3:1, HCl concentration = 1.2 mol/L, the molar ratio of APS to oPD = 1:1. The apparent first-order rate constant *k*_*app*_ of PoPD/TiO_2_ nanocomposite was 0.0098 min^-1^, which is 6 times higher than TiO_2_ (0.0016 min^-1^). Meanwhile, the PoPD/TiO_2_ nanocomposites showed excellent photocatalytic stability, and the photocatalytic stability was depended on the stability of structure. At last, the photocatalytic mechanism of POPD/TiO_2_ nanocomposites was also proposed based on the synergetic effect between TiO_2_ and PoPD.

## Introduction

Semiconductor as a high-profile photocatalyst has been widely applied in various areas ranging from solar cell to water pollution control [1–3]. Recent research showed that TiO_2_-based heterogeneous photocatalytic oxidation technologies are still the most promising methods because of their outstanding oxidative power and stability [4–6]. During recent decades, enormous efforts have been devoted to developing a series of semiconductor photocatalysts, such as TiO_2_, ZnO, CdS, and so on [7–10]. However, slow reaction rate, poor solar efficiency, the low quantum efficiency, the critical drawback of photocorrosion, and secondary pollution on the environment impaired their applications to a great extent [11–12]. To eliminate these drawbacks, many attempts have been carried out to modify surface of TiO_2_, such as doping, metal deposition, compound semiconductor, and conducting polymer modifying [13].

Recently, the properties of conducting polymer in electron-transfer processes have been widely studied to show they can efficiently arouse a rapid photoinduced charge separation and a relatively slow charge recombination [14]. Zhang Hao et al. and Lin Yangming et al. prepared PANI/TiO_2_ nanocomposites and they found the as-prepared samples have enhanced photocatalytic activity under visible light [15–16]. Li Xueyan et al. and Wang Desong et al. prepared PANI/TiO_2_ and PPY/TiO_2_ nanocomposites via ‘in situ’ oxidative polymerization method and then indicated composite photocatalyst with excellent photocatalytic performance was attributed to the sensitizing effect of conducting polymer, and the synergetic effect between conducting polymer and TiO_2_ [17–18].

In addition to PANI and PPY, poly-o-phenylenediamine (PoPD) was also paid attehtion to the research of conducting polymer modified TiO_2_ to improve its photocatalytic performance [19]. As a typical conducting polymer, PoPD has attracted considerable attention since its discovery. Taking advantage of the unique electrical, optical and photoelectric properties of PoPD, we expect that the combination of PoPD with TiO_2_ may induce an interesting charge transfer and thus enhance the photocatalytic activity of TiO_2_ under visible light irradiation [20]. However, the photocatalytic activity enhanced mechanism has not been studied. The photocatalytic process of PoPD/TiO_2_ involves a primary reaction process that generates holes and electrons and a secondary reaction process that generates reactive oxygen species [21]. Therefore, achieving a quantitative estimate of the contributions of PoPD under the primary and secondary reaction processes is important [22].

However, in most cases, the photocatalytic degradation of organic pollutants was mainly for organic dyestuff, and little research was performed on phenols, highly toxic and carcinogenic compounds, meanwhile the mechanism of PoPD/TiO_2_ nanocomposite photocatalyst under the visible light has not been convincingly explained [23].

In this study, PoPD/TiO_2_ nanocomposite was successfully synthesized via ‘in situ’ oxidative polymerization method. The modified photocatalysts were characterized by BET, scanning electron microscopy (SEM), transmission electron microscopy (TEM), X-ray diffraction (XRD), Fourier-transform infrarad spectra (FT-IR), thermogravimrtic analysis (TGA), X-ray photoelectron spectroscopy (XPS), Ultraviolet-visible diffuse reflectance spectrum (UV-Vis DRS) and Photocurrent Test. The photocatalytic degradation of methylene blue (MB) was chosen as a model reaction to evaluate the photocatalytic activities of TiO_2_ and PoPD/TiO_2_, results indicated that the PoPD/TiO_2_ nanocomposites showed excellent photocatalytic activity and stability. Meanwhile, the photocatalytic mechanism of POPD/TiO_2_ nanocomposites was also proposed based on the synergetic effect between TiO_2_ and PoPD. It hoped our works could provide valuable information on the synthesis and application of conducting polymer modified semiconductor.

## Materials and methods

### Materials

The o-phenylenediamine (oPD), ammonium persulfate (APS), sulfuric acid (H_2_SO_4_) and sodium hydroxide (NaOH) were purchased from Tianjin Kermel Chemical Reagent Co., Ltd. The anatase TiO_2_ was purchased from Aladdin Chemical Reagent Co., Ltd. The ethyl alcohol was purchased from Tianjin Fuyu Fine Chemical Co., Ltd. The hydrochloric acid was purchased from Sinopharm Chemical Reagent Co., Ltd. The methylene blue (MB) was purchased from Tianjin Guangcheng Chemical Reagent Co., Ltd. All chemicals are analytical grade without further purification. Deionized water was used for the synthesis of all solutions.

### Sample synthesis

The typical synthesis of PoPD/TiO_2_ nanocomposite photocatalyst was as follows: An appropriate amount of oPD was dissolved in 90 ml 1.2 mol/L hydrochloric acid solution, with 0.256 g anatase TiO_2_ adding. The solution was ultrasonic cleaning 15 min to mixing uniformity. After dissolving, the solution was labeled A. An appropriate amount of APS was dissolved in 30 ml 1.2 mol/L hydrochloric acid solution, the solution was labeled B. The solution A was transferred to a 250 ml round-bottom flask, magneton was added, and the solution was stirred with a magnetic stirrer. The solution B was transferred to a 100 ml constant pressure funnel and then dropped in solution A at about 1drop/second with stirring. The reaction was continued with 24 h at room temperature. The final products were filtered and washed with deionized water and ethanol and dried at 80°C for several hours in a vacuum oven.

### Characterization

The surface texture of TiO_2_ and PoPD/TiO_2_ nanocomposite was examined by N_2_ adsorption at 77 K (Quantachrome instruments Quadrasorb SI). The specific surface area was calculated from the N_2_ adsorption isotherm using the BET equation. Scanning electron microscope (SEM) patterns were performed on a QUANTA F250 cold field emission scanning electron microscope. Transmission electron microscopy (TEM) patterns were performed on a FEI Tecnai G2 20 transmission electron microscopy. X-Ray Diffraction (XRD) patterns were recorded on a Bruker D8 Advance X-ray diffractometer with Cu Kɑ radiation. Fourier-transform infrarad spectra (FT-IR) of the samples were recorded on Vertex 70 spectrometer in a range from 4000 to 400 cm^-1^. Thermogravimetric analysis (TGA) of all of the samples were performed with a Q500 thermal analysis instrument (TA instruments Co., Ltd.). The samples were heated from 35 to 800°C at a rate of 10°C min^-1^ in air. X-ray photoelectron spectroscopy (XPS) measurements were performed using a Thermo ESCALAB 250Xi system with an Al Kα X-ray source. All of the binding energies were referenced to the C1s peak at 284.8 eV for the surface adventitious carbon. Ultraviolet-visible diffuse reflectance spectrum (UV-Vis DRS) were detected by an UV-2550PC ultraviolet and visible spectrophotometerwith BaSO_4_ as the background ranging from 200 to 800 nm. Photocurrent test was measured using a CHI660D VersaSTAT. The TiO_2_ and PoPD/TiO_2_ nanocomposites were deposited as a film on a 2 cm × 2 cm indium-tin-oxide conducting glass to obtain the working electrode. The saturated calomel electrode and Pt electrode served as the reference electrode and the counter electrode, respectively. The electrolyte was 0.1 mol/L Na_2_SO_4_ solution.

### Photocatalytic activity test

The photocatalytic activity was evaluated by the decomposition of MB under visible light (λ >450 nm). The visible light was obtained by a 1000W xenon lamp (XPA-II Photochemical Reactions Instrument) with a 400 nm cutoff filter to ensure the desired irradiation light. Aqueous suspensions of MB (30 mL, 10 mg/L) were placed in a quartz tube, and 30 mg of PoPD/TiO_2_ nanocomposite photocatalyst were added. Prior to irradiation, the suspensions were magnetically stirred in darkness for about 3 h. The suspensions were kept under constant air-equilibrated conditions before and during illumination. At certain time intervals, 1 mL liquor was sampled and centrifuged to remove the particles. The filtrates were analyzed by recording variations of the maximum absorption band (664 nm for MB) using a UV-2550PC ultraviolet and visible spectrophotometer. To evaluate the accuracy the PoPD/TiO_2_ nanocomposite photocatalyst, the photocatalytic process was reused four times to degrade MB under visible light.

## Results

### Physicochemical properties of PoPD/TiO_2_ nanocomposite

**Fig 1:**
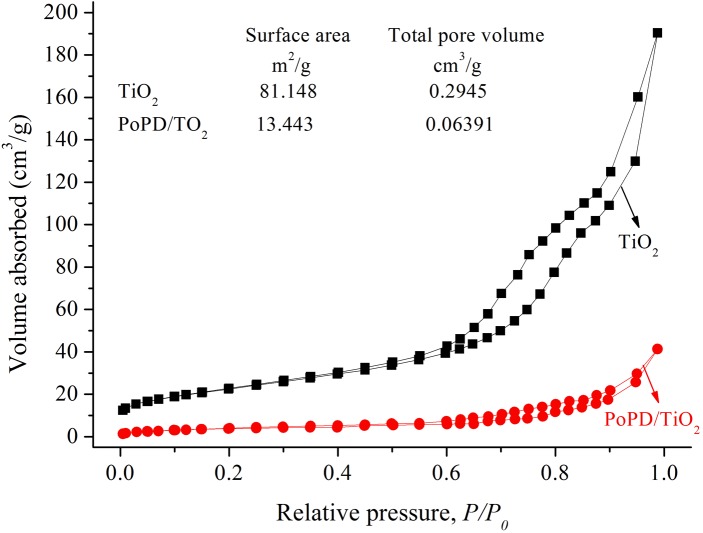
N_2_ adsorption and desorption isotherms at 77 K onTiO_2_ and PoPD/TiO_2_.

It was well-known that the photocatalytic activity was governed by various factors such as surface area, phase structure, interfacial charge transfer, and separation efficiency of photoinduced electrons and holes. The adsorption and desorption isotherms of N_2_ at 77 K on TiO_2_ and PoPD/TiO_2_ nanocomposites, are shown in Fig 1. Like the anatase TiO_2_, the PoPD/TiO_2_ nanocomposite also displays a Type II isotherm characteristic of a mesoporous material [24]. Clearly the total pore volume and surface area of PoPD/TiO_2_ nanocomposite are much less than those of TiO_2_. However, the PoPD/TiO_2_ nanocomposite showed the higher photocatalytic activity than TiO_2_, indicating that the surface area of photocatalyst is only an index to character the physicochemical properties, not the decisive index to ensure the photocatalytic activity.

**Fig 2:**
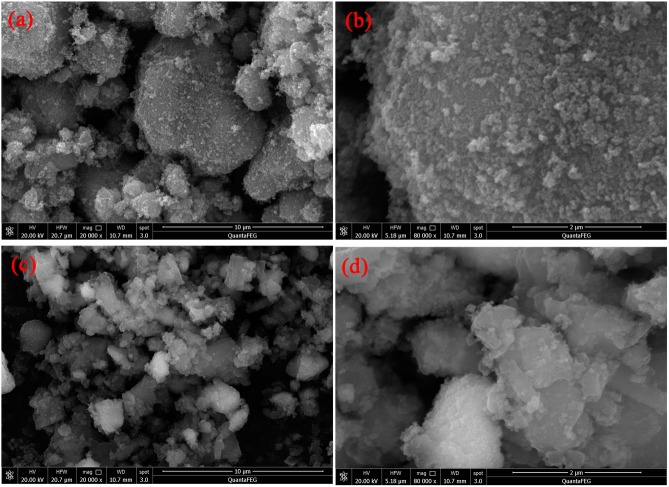
**SEM images of (a-b) TiO_2_ and (c-d) PoPD/TiO_2_ nanocomposites**.

Fig 2 shows the SEM images of TiO_2_ and PoPD/TiO_2_ nanocomposites. Compared with TiO_2_ (Fig 2A and 2B), PoPD/TiO_2_ nanocomposite (Fig 2C and 2D) possessed of more smooth interface, indicating that TiO_2_ and PoPD layer formed the core-shell structure. The grain size was about 30–50 nm, and SEM patterns of TiO_2_ and PoPD/TiO_2_ showed no change before and after modification by PoPD, indicating that the deposited PoPD layer was very thin. There were agglomeration phenomena discernable because the grain size was small and the stong acting force (Van der Waals' Force and electrostatic attraction). Compared with TiO_2_, PoPD/TiO_2_ composite photocatalyst possessed a low dispersion degree and obvious agglomeration phenomenon because of the oxidation polymerization reaction and PoPD conglutination.

**Fig 3:**
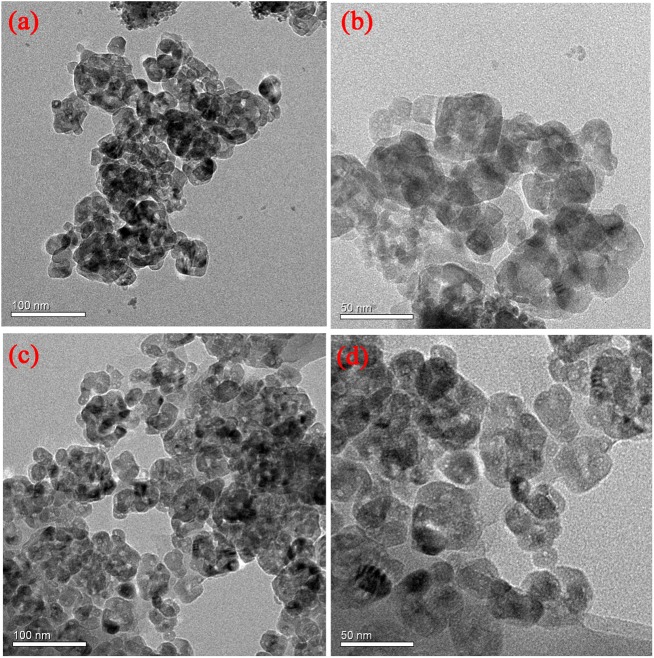
**TEM images of (a-b) TiO_2_ and (c-d) PoPD/TiO_2_ nanocomposites**.

Fig 3 shows the TEM images of TiO_2_ and PoPD/TiO_2_ nanocomposites. From the results shown in Fig 3A, 3B, 3C and 3D, there were agglomeration phenomena of PoPD/TiO_2_ nanocomposite observable because of high surface energy. The grain size of PoPD/TiO_2_ nanocomposite observed in TEM images was about 30–50 nm, and the result was consistent with SEM images.

**Fig 4:**
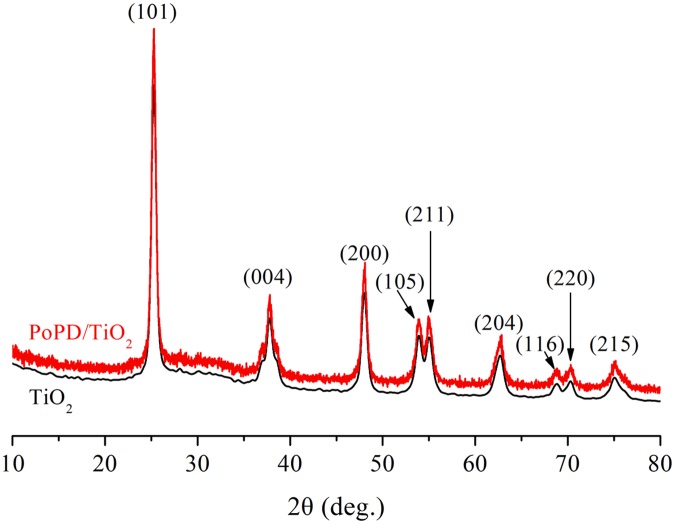
XRD pattern of TiO_2_ and PoPD/TiO_2_ nanocomposites.

Fig 4 shows the XRD spectra of TiO_2_ and PoPD/TiO_2_ nanocomposites. The peaks at 2θ values of 25.3°, 37.8°, 48.0°, 53.9°, 55.1°, 62.7° 68.8°, 70.3°, and 75.0° can be indexed to (101), (004), (200), (105), (211), (204), (116), (220), and (215) faces of anatase TiO_2_, respectively. It is obvious that the PoPD/TiO_2_ nanocomposite has not change in peak positions and shapes compared with the pure TiO_2_, indicating that the presence of PoPD does not impact on the lattice structure of TiO_2_.

The grain size can be calculated by the following Scherrer Equation:

$$D=\frac{K\lambda }{\left(\cos\theta •B_{1/2}\right)}$$ (1)

where *D* represents grain size, *K* is the Scherrer constant of diffraction peak and the value of anatase TiO_2_ crystal is 0.89, *λ* represents the wavelength of X ray, *B*_*1/2*_ is full width at half maximum of diffraction peak, *θ* is the Bragg Diffraction Angle. Based on the (101) face main peak at 2θ value of 25.3° of PoPD/TiO_2_ photocatalyst, the calculated grain size was 44.6 nm. Comparing with calculated the grain size of anatase TiO_2_ crystal (44.2 nm), the presence of PoPD does not impact on the grain size of TiO_2_.

**Fig 5:**
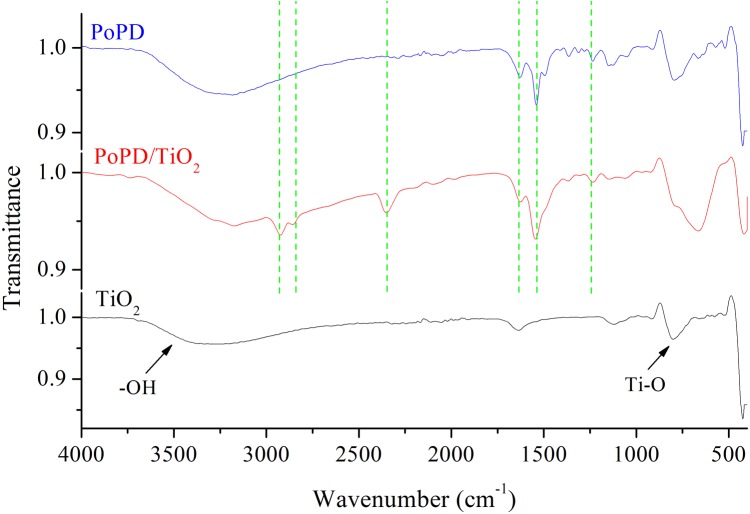
FT-IR spectra of TiO_2_, PoPD, and PoPD/TiO_2_ nanocomposites.

The FT-IR spectra of TiO_2_, PoPD, and PoPD/TiO_2_ are shown in Fig 5. The main characteristic bands of PoPD are assigned as follows: the peak at 1629 cm^-1^ is associated with C = N stretching vibration, and the strong absorption band at 1523 cm^-1^ is ascribed to the C = C stretching vibrations in the benzene ring. The weak peaks at 1328 cm^-1^ and 1238 cm^-1^ are correspondingly assigned to the = C-N stretching on the benzene ring. The FT-IR spectrum of the PoPD/TiO_2_ contains the same main characteristic bands as that of PoPD but with a shift to higher wavenumbers [25]. The results show that there is a strong interaction between PoPD and the TiO_2_ nanoparticles (2350 cm^-1^, 2850 cm^-1^, and 2925 cm^-1^), and the PoPD deposits and forms a shell on the surface of the TiO_2_ nanoparticles. The deposition of PoPD on the surface of the TiO_2_ nanoparticles not only constrains the motion of the PoPD chains but also restricts the vibration mode in the PoPD molecule. It can be observed that the characteristic band of TiO_2_ at 745 cm^-1^ (Ti-O) occurred in the PoPD/TiO_2_ nanocomposite and the band is so wide that it hides the figure peak in the PoPD/TiO_2_ nanocomposite.

**Fig 6:**
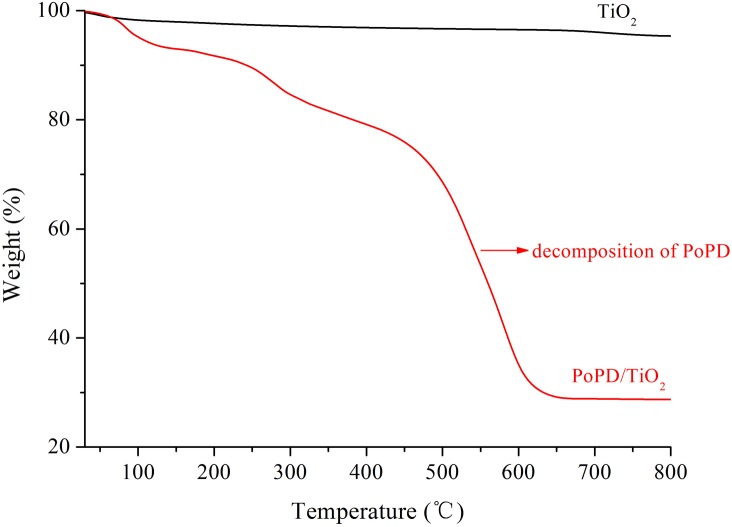
**TGA curves of (a) TiO_2_ and (b) PoPD/TiO_2_ nanocomposites**.

The thermal behavior of TiO_2_ and PoPD/TiO_2_ was investigated by TGA, and the results are shown in Fig 6. In Fig 6, curve (a) indicates that TiO_2_ is very stable in air, and no decomposition occurred in the 30–800°C range. The thermogravimetric curve of PoPD/TiO_2_ is shown in Fig 6(B). The first weight loss was observed at 100°C owing to desorption of the water that was absorbed on PoPD/TiO_2_ nanocomposite This curve also indicates that a sharp weight loss occurs at approximately 450°C and continues up to 600°C. This weight loss was due to decomposition of PoPD.

**Fig 7:**
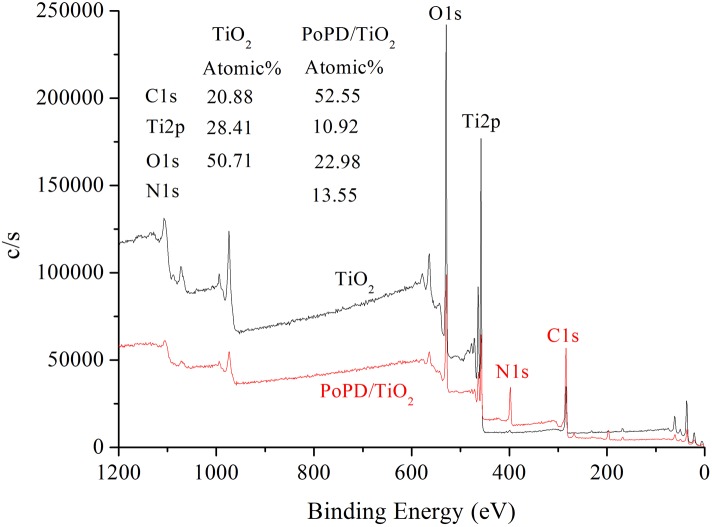
XPS spectra of TiO_2_ and PoPD/TiO_2_ nanocomposites.

X-ray photoelectron spectroscopy (XPS) is an important tool for studying the electronic structure of condensed matter and is widely used for quantitative surface analysis. According to the XPS survey spectra (Fig 7) of TiO_2_ and PoPD/TiO_2_, Ti and O were present in TiO_2_ based on the two peaks at binding energies of 458.5 and 529.8 eV. In addition, the C, O, Ti and N elements existed in the TiO_2_ based on the four peaks with binding energies of 284.8, 529.8, 458.5 and 400.3 eV, which are related to C1s, O1s, Ti2p and N1s, respectively [26]. The atomic percentages of C, O, Ti and N were 52.55%, 22.98%, 10.92% and 13.55%, respectively, suggesting that PoPD exists on the TiO_2_ surface.

**Fig 8:**
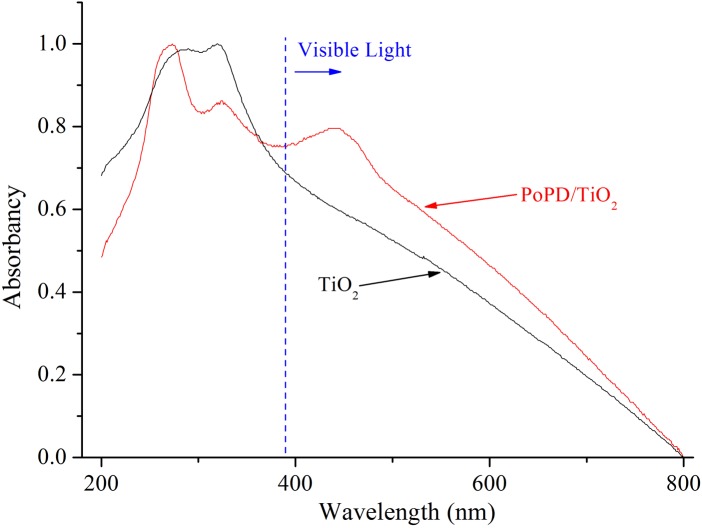
UV-Vis DRS of TiO_2_ and PoPD/TiO_2_ nanocomposites.

To obtain information about response to ultraviolet light and visible light of the samples, TiO_2_ and PoPD/TiO_2_ nanocomposites were characterized by UV-Vis DRS. As shown in Fig 8, it can be observed that both TiO_2_ and PoPD/TiO_2_ had strong responses to UV light, but PoPD/TiO_2_ had the stronger responses to visible light. Meanwhile, there was an absorption peak at 460 nm of PoPD/TiO_2_. The results not only proved the existence of PoPD on the TiO_2_ surface, but also explained the reason why PoPD/TiO_2_ had the higher photocatalytic performance than TiO_2_ under visible light [27].

**Fig 9:**
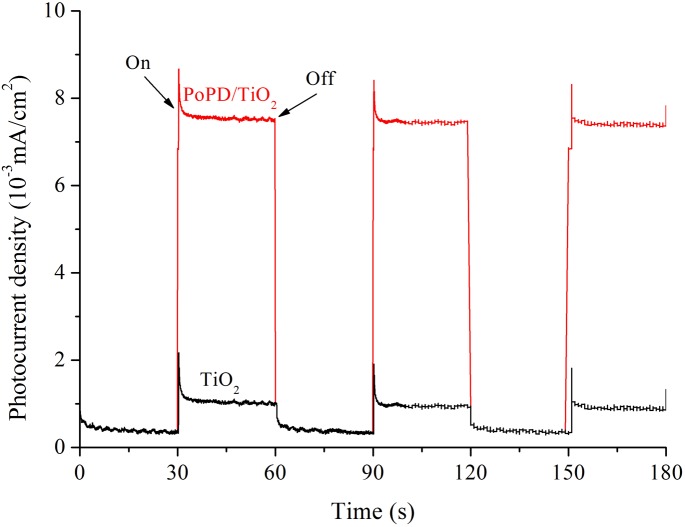
Photocurrent density of TiO_2_ and PoPD/TiO_2_ nanocomposites under visible light irradiation.

To obtain information of photoelectric property, TiO_2_ and PoPD/TiO_2_ nanocomposites were characterized by Photocurrent Test. As shown in Fig 9, the photocurrent density of TiO_2_ was low (about 1μA/cm^2^) under visible light because the pure TiO_2_ band gap was about 3.2 eV, but photocurrent density of PoPD/TiO_2_ nanocomposite under visible light was 7 times as high as pure TiO_2_. The results indicated that there was a heterostructure between TiO_2_ and PoPD to possessing the strong responses to visible light and producing abundant hole-electron pairs. The PoPD had excellent electrical conductivity to transfer free electrons from VB to CB, showing the PoPD/TiO_2_ had higher photocurrent density and photocatalytic performance than TiO_2_. Similar results were also obtained with the research of Liao et al about photonic crystal coupled TiO_2_/polymer hybrid for efficient photocatalysis under visible light irradiation [28–29]. The results proved that the combination of TiO_2_ and PoPD was an effective way to improve photocatalytic activity.

### Photocatalytic activity of PoPD/TiO_2_ nanocomposite

The photocatalytic performance of PoPD/TiO_2_ nanocomposites for liquid-phase degradation of MB has been measured for the molar ratio of oPD to TiO_2_, the concentration of hydrochloric acid, and the molar ratio of APS to oPD. MB has a maximum absorption at about 664 nm.

**Fig 10:**
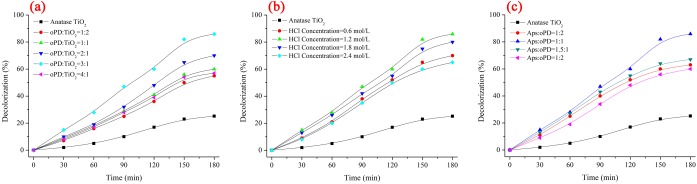
**Results of degrading MB using different (a) molar ratios of oPD to TiO_2_ nanocomposites, (b) the concentration of hydrochloric acid, and (c) the molar ratio of APS to oPD**.

The influences of molar ratios of oPD to TiO_2_ in the oxidative polymerization reaction to PoPD/TiO_2_ photocatalytic performance are shown in Fig 10(A). The influences of the concentration of hydrochloric acid in the oxidative polymerization reaction to PoPD/TiO_2_ photocatalytic performance are shown in Fig 10(B). The influences of the molar ratios of APS to oPD in the oxidative polymerization reaction are shown in Fig 10(C). The kinetics plots are shown by apparent first-order linear transform -ln(*C/C*_*0*_) = *k*_*app*_*t*. The activity of TiO_2_ and PoPD/TiO_2_ nanocomposites can be evaluated by comparing the apparent first-order rate constants (*k*_*app*_) list in Table 1.

**Table 1:** Apparent first-order rate constants (*k*_*app*_) of MB degradation and linear regression coefficients from -ln(*C/C*_*0*_) = *k*_*app*_*t*.

Samples		-ln(*C/C*_*0*_) = *k*_*app*_*t*	*k*_*app*_	R^2^	$\frac{k_{P/T}}{k_{T}}$^b^
	TiO_2_	-ln(*C/C*_*0*_) = *0*.*0016t*	0.0016	0.9443	
oPD:TiO_2_	1:2	-ln(*C/C*_*0*_) = *0*.*0041t*	0.0041	0.9538	2.56
	1:1	-ln(*C/C*_*0*_) *= 0*.*0048t*	0.0048	0.9556	3.00
	2:1	-ln(*C/C*_*0*_) *= 0*.*0061t*	0.0061	0.9303	3.81
	3:1^a^	-ln(*C/C*_*0*_*) = 0*.*0098t*	0.0098	0.9108	6.13
	4:1	-ln(*C/C*_*0*_) = 0.0045*t*	0.0045	0.9559	2.81
HCl	0.6 mol/L	-ln(*C/C*_*0*_) = *0*.*0064t*	0.0064	0.9600	4.00
	1.2 mol/L^a^	-ln(*C/C*_*0*_) = *0*.*0098t*	0.0098	0.9108	6.13
	1.8 mol/L	-ln(*C/C*_*0*_) = *0*.*0081t*	0.0081	0.9297	5.06
	2.4 mol/L	-ln(*C/C*_*0*_) = *0*.*0057t*	0.0057	0.9676	3.56
Aps:oPD	1:2	-ln(*C/C*_*0*_) = *0*.*0058t*	0.0058	0.9858	3.63
	1:1^a^	-ln(*C/C*_*0*_) = *0*.*0098t*	0.0098	0.9108	6.13
	1.5:1	-ln(*C/C*_*0*_) = *0*.*0064t*	0.0064	0.9872	4.00
	2:1	-ln(*C/C*_*0*_) = *0*.*0051t*	0.0061	0.9754	3.81

a: The optimal preparation condition is oPD:TiO_2_ = 3:1, HCl concentration = 1.2 mol/L, and Aps:oPD = 1:1.

b: The specific value between *k*_*app*_ of PoPD/TiO_2_ (*k*_*P/T*_) and *k*_*app*_ of TiO_2_ (*k*_*T*_).

As can be seen from the Fig 10 and Table 1, the photocatalytic degrading efficiency was increased with the molar ratio of oPD to TiO_2_ increased, but it was decreased when the molar ratio of oPD to TiO_2_ was over 3:1. The photocatalytic degrading efficiency was increased with the concentration of hydrochloric acid from 0.6 mol/L to 1.2 mol/L, the photocatalytic degrading efficiency was resembled with the concentration of hydrochloric acid from 1.2 mol/L to 1.8 mol/L, and the photocatalytic degrading efficiency was decreased with the concentration of hydrochloric acid from 1.8 mol/L to 2.4 mol/L. When the molar ratio of APS to oPD was over 1:1, the photocatalytic performance of PoPD/TiO_2_ composite photocatalyst decreased with the molar ratio of APS to oPD increased. So the optimal preparation condition was the molar ratio of oPD to TiO_2_ 3:1, the concentration of hydrochloric acid 1.2 mol/L, and the molar ratio of APS to oPD 1:1.

### Photocatalytic stability of PoPD/TiO_2_ nanocomposite

**Fig 11:**
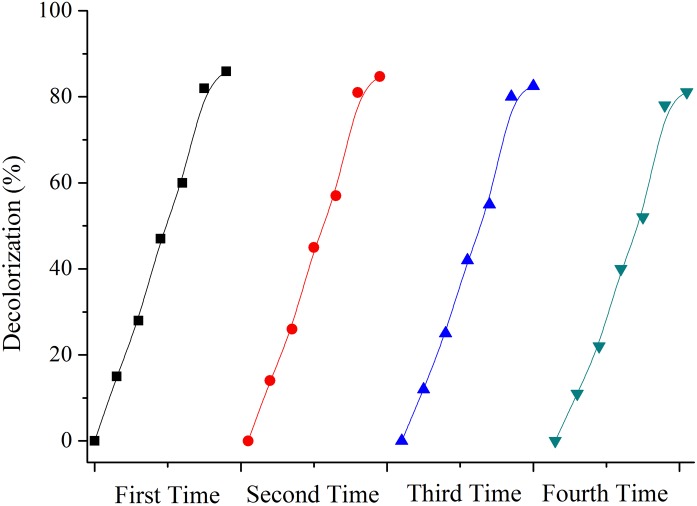
Photocatalytic degradation rate of MB with PoPD/TiO_2_ nanocomposites in different recycling time.

To obtain information about the stability of the PoPD/TiO_2_ nanocomposite, the recycling experiments were finished. From the results shown in Fig 11, after four times run of degradation reaction, the photocatalytic decolourization ratio of MB was decreased from 85.9% to 81.1% after 4 h irradiation under visible light. So the prepared PoPD/TiO_2_ nanocomposite possessed excellent photocatalytic stability.

## Discussion

### Preparation condition and photocatalytic activity of PoPD/TiO_2_ nanocomposite

Considered the pure anatase TiO_2_ had no response to visible light, the PoPD had response to visible light and excited electrons from valence band (VB) to conduction band (CB), the ·OH free radical were produced in PoPD/TiO_2_ to destain and degrade MB.

When the molar ratio of oPD to TiO_2_ was low, with the molar ratio of oPD to TiO_2_ increasing, the thickness of deposited PoPD on TiO_2_ surface was increasing, so the producing electron-hole pairs accumulated, and the photocatalytic performance was stronger. However, when the molar ratio of oPD to TiO_2_ continued to increase, the thickness of deposited PoPD on the TiO_2_ surface was too thick to influence the transmission of producing electron, so the photocatalytic performance of PoPD/TiO_2_ nanocomposite decreased.

It was well-known that hydrochloric acid not only provides the acidity in oxidative polymerization reaction, but also the electrolytic Cl^-^ can mix into intramembrane to neutralize the positive charge [30]. The Cl^-^ mixing can avail the charge delocalization in the PoPD molecular chain to enhance the electrical conductivity of PoPD. However, when the concentration of hydrochloric acid was too high, the mixing amount was troppo to influence the contact between oPD molecules. Then the results led to the molecular chain shortening and electrical conductivity decreasing, which led to the photocatalytic performance of PoPD/TiO_2_ decreased.

When the molar ratio of APS to oPD was low in the PoPD oxidative polymerization reaction, considered lack of the active sites for the reaction, the oxidative polymerization reaction was inclined to produce macromolecule PoPD, so the conductivity and productivity of PoPD increased with the amount of APS increased. When the molar ratio of APS to oPD was too high in the PoPD oxidative polymerization reaction, not only the superfluous active sites for the reaction were led to the disadvantage of producing macromolecule PoPD, but also the superfluous APS oxidated the main molecular chain to break the conjugated structure. So the reaction was led to a decrease in the conductivity and productivity of PoPD, and the photocatalytic performance of PoPD/TiO_2_ decreased [31].

### Photocatalytic stability of PoPD/TiO_2_ nanocomposite

**Fig 12:**
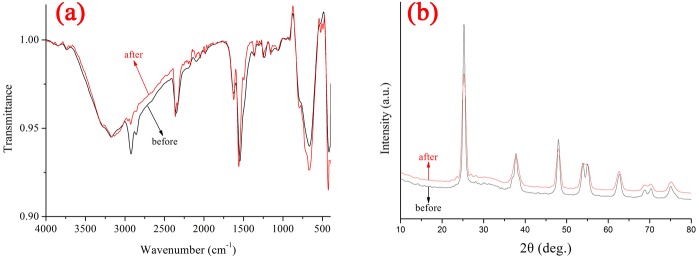
**(a) FT-IR spectra and (b) XRD patterns of PoPD/TiO_2_ nanocomposites before and after photocatalytic reaction**.

In order to ensure the photocatalytic stability of PoPD/TiO2 nanocomposite, the FT-IR spectra and XRD patterns before and after reaction were redorded as shown in Fig 12. It is found that the shape of FT-IR and XRD after photocatalytic reaction is similiar to that before reaction. It indicates that the structure of PoPD/TiO_2_ nanocomposite does not change during the photocatalytic process, and the stability of photocatalytic activity is depended on the stability of structure.

### Photocatalytic mechanism

**Fig 13:**
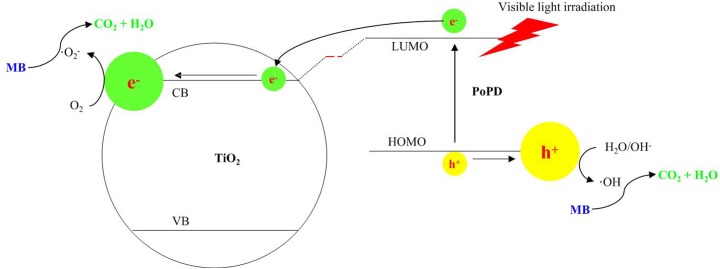
Photocatalytic mechanism of PoPD/TiO_2_ nanocomposites to enhance photocatalytic activity under visible light irradiation.

The synergetic effect between TiO_2_ and PoPD on the photocatalytic degradation of MB exists clearly for all the PoPD/TiO_2_ nanocomposites. The mechansim of PoPD on the activity of the PoPD/TiO_2_ nanocomposites can be explained as photosensitizer (Fig 13). It was well-known that TiO_2_ had the special energy-band structure which means including valence band (VB) to conduction band (CB). The band-gap energy of anatase TiO_2_ was 3.2 eV, indicating that it only had response to small amount light (λ>387 nm). Because PoPD has charge-transfer excitation-like transition from the Highest Occupied Molecular Orbital (HOMO) to the Lowest Unoccupied Molecular Orbital (LUMO) can lead to that itself excited photogenerated electrons transfer to the CB of TiO_2_ and it accepts the holes from the VB of TiO_2_. On the one hand the photogenerated electrons were transferred to CB to produce e^-^_CB_, on the other hand there were holes (h^+^) in VB after electrons transferred to PoPD. The free electrons e^-^_CB_ reacted with O_2_ to produce superoxide radical ·O_2_^-^, and holes h^+^ reacted with OH^-^ and H_2_O to produce hydroxyl radical ·OH. And the reactive oxygen species are responsible for the degradation of MB [32–34].

The photocatalytic mechanism of PoPD/TiO_2_ nanocomposites under visible light was expounded as following [35–36].

$$PoPD/TiO_{2}\overset{vis-light}{\to }PoPD^{+}/TiO_{2}+e^{-}$$ (2)

$$e^{-}+O_{2}\to •O_{2}^{-}$$ (3)

$$PoPD^{+}/TiO_{2}\to PoPD/TiO_{2}+h^{+}$$ (4)

$$h^{+}+OH^{-}\to •OH$$ (5)

$$h^{+}+H_{2}O\to •OH+H^{+}$$ (6)

## Conclusions

The PoPD/TiO_2_ nanocomposites were prepared via ‘in situ’ oxidative polymerization method using APS as oxidant, oPD as monomers, and anatase TiO_2_ particles as titanium source. The optimal preparation conditions included that the molar ratio of oPD to TiO_2_ was 3:1, hydrochloric acid concentration was 1.2 mol/L, the molar ratio of APS to oPD was 1:1.

The photocatalysts were characterized by BET, XRD, SEM, TEM, UV-VIS DRS and Photocurrent Test, and the results showed that the PoPD exists on the surface of TiO_2_, the presence of PoPD do not impact on the lattice structure and grain size of TiO_2_, and the presence of PoPD enhances the visible response and photoelectric property.

The photocatalytic degradation of methylene blue (MB) was chosen as a model reaction to evaluate the photocatalytic activities of TiO_2_ and PoPD/TiO_2_. The decolorization ratio of MB using PoPD/TiO_2_ nanocomposite prepared with optimal preparation condition was 85.9% (apparent first-order rate constant *k*_*app*_ was 0.0098 min^-1^), which is higher than using TiO_2_ Photocatalyst (decolorization ratio of MB was 25.2%; apparent first-order tare constant *k*_*app*_ was 0.0016 min^-1^). Meanwhile, the PoPD/TiO_2_ nanocomposites showed excellent photocatalytic stability, and the photocatalytic stability was depended on the stability of structure via the FT-IR spectra and XRD patterns before and after photocatalytic reaction. At last, the photocatalytic mechanism of POPD/TiO_2_ nanocomposites was also proposed based on the synergetic effect between TiO_2_ and PoPD.
